# Inflammation-Induced Cell Proliferation Potentiates DNA Damage-Induced Mutations *In Vivo*

**DOI:** 10.1371/journal.pgen.1004901

**Published:** 2015-02-03

**Authors:** Orsolya Kiraly, Guanyu Gong, Werner Olipitz, Sureshkumar Muthupalani, Bevin P. Engelward

**Affiliations:** 1 Department of Biological Engineering, Massachusetts Institute of Technology, Cambridge, Massachusetts, United States of America; 2 Singapore–MIT Alliance for Research and Technology, Singapore; 3 Division of Comparative Medicine, Massachusetts Institute of Technology, Cambridge, Massachusetts, United States of America; University of Washington, UNITED STATES

## Abstract

Mutations are a critical driver of cancer initiation. While extensive studies have focused on exposure-induced mutations, few studies have explored the importance of tissue physiology as a modulator of mutation susceptibility *in vivo*. Of particular interest is inflammation, a known cancer risk factor relevant to chronic inflammatory diseases and pathogen-induced inflammation. Here, we used the fluorescent yellow direct repeat (FYDR) mice that harbor a reporter to detect misalignments during homologous recombination (HR), an important class of mutations. FYDR mice were exposed to cerulein, a potent inducer of pancreatic inflammation. We show that inflammation induces DSBs (γH2AX foci) and that several days later there is an increase in cell proliferation. While isolated bouts of inflammation did not induce HR, overlap between inflammation-induced DNA damage and inflammation-induced cell proliferation induced HR significantly. To study exogenously-induced DNA damage, animals were exposed to methylnitrosourea, a model alkylating agent that creates DNA lesions relevant to both environmental exposures and cancer chemotherapy. We found that exposure to alkylation damage induces HR, and importantly, that inflammation-induced cell proliferation and alkylation induce HR in a synergistic fashion. Taken together, these results show that, during an acute bout of inflammation, there is a kinetic barrier separating DNA damage from cell proliferation that protects against mutations, and that inflammation-induced cell proliferation greatly potentiates exposure-induced mutations. These studies demonstrate a fundamental mechanism by which inflammation can act synergistically with DNA damage to induce mutations that drive cancer and cancer recurrence.

## Author Summary

People with chronic inflammatory conditions have a markedly increased risk for cancer. In addition, many cancers have an inflammatory microenvironment that promotes tumor growth. Here, we show that inflammatory infiltration synergizes with tissue regeneration to induce DNA sequence rearrangements *in vivo*. Chronically inflamed issues that are continuously regenerating are thus at an increased risk for mutagenesis and malignant transformation. Further, rapidly dividing tumor cells in an inflammatory microenvironment can also acquire mutations, which have been shown to contribute to drug resistance and disease recurrence. Finally, inflammation-induced tissue regeneration sensitizes tissues to DNA damaging environmental exposures and chemotherapeutics. The work described here thus increases our understanding of how inflammation leads to genetic changes that drive cancer formation and recurrence.

## Introduction

Effective strategies for preventing and treating cancer depend not only upon understanding genetic and exposure-induced factors, but also physiological factors that drive disease. DNA damage, caused by endogenous metabolites and exogenous agents, promotes mutations, a key driver of phenotypic changes that potentiate metastasis and enable recurrence after treatment [1]. While significant progress has been made in terms of understanding how genes and exposures modulate the risk of mutations, relatively little is known about the potential role of tissue physiology in modulating the risk of mutations *in vivo*. Of particular interest is the inflammatory state, a critical cancer risk factor that is associated with sweeping changes in tissue architecture due to immune cell infiltration and associated changes in the levels of cytokines and reactive oxygen and nitrogen species (RONS) [2–4]. Inflammation is a well-established tumor promoter that contributes to cancer growth, angiogenesis, and resistance to apoptosis [2,5]. In addition to the role of inflammation in cancer progression, it is increasingly recognized that inflammation-induced DNA damage may also drive mutations that contribute to both initiation and progression [3,6]. With recent advances that enable analysis of key factors that impact the risk of mutation [7], here, we set out to determine how interactions between DNA damage and inflammation-induced physiological changes impact the risk of mutations *in vivo*.

It has long been thought that it is the convergence of conditions that induce DNA damage and cell division simultaneously that is a key driver of inflammation-induced mutations [8–11]. Nevertheless, studies that directly query the combined effect of RONS-induced DNA damage and cell division are lacking, both *in vitro* and *in vivo*. Importantly, the same proposed mechanism for synergy between cell division and endogenous RONS applies to exogenous DNA damaging agents. In the clinic, virtually all cancer patients are exposed to high levels of DNA damage when treated with radiation and/or chemotherapy, for which DNA damage is often critical to the mode of action. It is well established that an increase in the mutation rate contributes to cancer promotion and drug resistance [12–15]. Therefore, understanding physiological factors that modulate susceptibility to therapy-induced mutations could open doors to strategies to reduce disease recurrence.

Pancreatic inflammation is a key risk factor for pancreatic cancer [11,16], one of the most deadly cancers; most patients who initially respond to radio-chemotherapy suffer relapse, such that only ∼5% of patients survive more than 5 years after diagnosis [17]. Inflammation-induced DNA damage potentially plays an important role in driving mutations that enable pancreatic cancer initiation and recurrence. During inflammation there are high levels of RONS, which can induce cytotoxic and mutagenic DNA lesions, including abasic sites, oxidized bases (e.g., 8oxoG), deaminated bases (e.g., uracil and hypoxanthine) and ethenoadenine (eA) [18,19]. In addition to base damage, RONS also induce DNA double strand breaks (DSBs). DSBs are among the most toxic of DNA lesions and they can also be potently mutagenic due to the potential loss of vast stretches of chromosomes if not accurately repaired [1,20].

Homologous recombination (HR) plays a critical role in preventing DSB-induced cytotoxicity by repairing DSBs during S/G_2_ [21]. To initiate repair, the DNA is resected by MRE11 and EXO1 to generate 3’ single-stranded overhangs [22–25]. BRCA2 then loads RAD51 onto the single-stranded DNA to form a nucleoprotein filament capable of homology searching and strand invasion [26–30]. The resulting D-loop enables the copying of sequence information that can then be processed by downstream proteins to complete the repair process [21]. While HR is effective for repair of two-ended DSBs, its most important role is in the repair of one-ended DSBs that arise when replication forks break down. Unlike two-ended DSBs, which can be repaired by alternative mechanisms, one-ended DSBs require HR for accurate sequence realignment and reinsertion of the broken DNA end. Inflammation induces single strand breaks and replication-blocking lesions, both of which promote replication fork breakdown. Furthermore, mutations in BRCA2 are genetic risk factor for pancreatic cancer [31], indicating that HR is indeed active in the pancreas [32]. Thus, RONS are predicted to create DSBs during pancreatitis, and HR can potentially repair inflammation-induced DSBs in the pancreas.

Ironically, while HR prevents cytotoxicity and is mostly accurate, HR carries a risk of sequence changes. Misalignments during HR promote large scale sequence rearrangements, such as deletions, duplications and translocations [33–35], and these HR-driven events have been observed in cancers [36,37]. Furthermore, HR between homologous chromosomes can also lead to loss of heterozygosity (LOH), a major mechanism for the inactivation of tumor suppressor genes. Indeed, studies with cultured cells have demonstrated that HR is the underlying cause of 30 to 70% of LOH events [38–40], and the importance of HR-driven LOH has also been demonstrated in tumors [41,42]. Finally, it has recently been shown that HR also promotes point mutations in mammalian cells, due to misincorporation during repair synthesis [43–48]. Taken together, it is now clear that virtually all cancers harbor one or more HR-driven sequence changes that promote initiation and progression.

**Figure 1:**
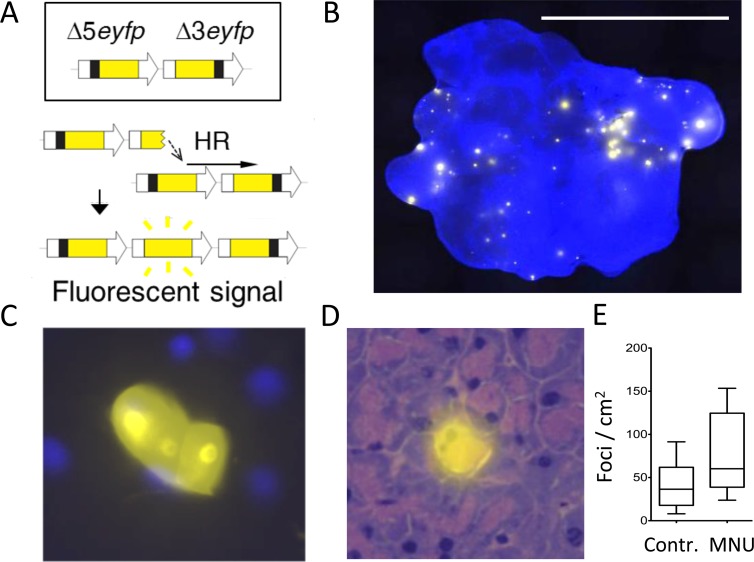
The FYDR mouse detects HR-derived sequence rearrangements *in situ* in intact tissue. (**A**) Schematic of the reconstitution of full-length EYFP coding sequence from two truncated copies through replication fork restart by HR. Note that the appearance of fluorescent signal indicates the gain of one repeat unit (a duplication). Arrows represent expression constructs. EYFP coding sequences are in yellow, promoter and polyadenylation signal sequences are in white, and deleted sequences are in black. Drawing is not to scale. (**B**) Representative image of a FYDR pancreas showing fluorescent foci detectable *in situ* in intact tissue. Freshly harvested, unfixed whole pancreas was counterstained with Hoechst, compressed to 0.5 mm and imaged under an epifluorescent microscope. Fluorescence is pseudocolored. Original magnification, ×1. Scale bar = 1 cm. (**C**) Cluster of recombinant cells at ×60 original magnification. Fluorescence is pseudocolored. (**D**) A recombinant pancreatic acinar cell identified by the overlay of EYFP fluorescence and H&E staining. Fluorescence is pseudocolored. Original magnification, ×40. (**E**) The model alkylating agent MNU induces HR in the pancreas. Mice received 25 mg/kg MNU i.p., and HR was evaluated 3 to 5 weeks after treatment. Frequencies of recombinant foci per cm^2^ tissue area are significantly greater in MNU-treated mice (n = 15) than in control mice (n = 16). Boxes show 25^th^ and 75^th^ percentiles, medians are indicated by horizontal lines. * *P* < 0.05 (Mann–Whitney *U*-test).

Given the importance of HR, we created a mouse model that enables the detection of HR *in vivo* (see ref. 7). The fluorescent yellow direct repeat (FYDR) mice harbor an integrated direct repeat comprised of two non-functional EYFP expression cassettes, wherein transfer of sequence information by HR from one cassette to the other can reconstitute full length sequence and give rise to fluorescence (Fig. 1A–D) [49]. The FYDR recombination substrate is designed to detect the major classes of HR events, including gene conversion (wherein sequence information is transferred from one duplex to the other), sister chromatid exchange (e.g., gene conversion with crossover) and replication fork repair (S1 Fig.) [50]. Importantly, FYDR fluorescence after replication fork repair indicates misalignment and transfer of sequence information during HR, and in some cases the gain of one repeat unit in the FYDR substrate (Fig. 1A). Given that all cells that are positive for fluorescence result from sequence misalignment and harbor a change in sequence information, the FYDR readout is indicative of mutation events. The FYDR mouse model thus affords key advances in studies of mutagenesis, since it became possible for the first time to visualize mutant cells that arise within intact tissues of adult animals [7].

Here, we have integrated approaches for visualization and quantification of DNA damage, cell proliferation, and mutation within intact tissues in order to learn about their interrelationships in the context of inflammation. We found that following controlled induction of acute inflammation, the timing for inflammation-induced DSBs is separate from the timing for cell proliferation, creating a protective kinetic barrier against potential synergy between DNA damage and cell division. Breaking this barrier by creating overlap between peak cell proliferation and the acute phase of inflammation causes a synergistic increase in HR-driven mutations. Furthermore, under conditions of inflammation-induced cell proliferation, there is a dramatic increase in susceptibility to mutations induced by exposure to an exogenous DNA damaging agent of a class that is present in environmental contaminants and also commonly used in the clinic. This work reveals the critical role that tissue physiology plays in mutation susceptibility and opens doors to new avenues of cancer prevention and treatment.

## Results

### FYDR mice enable studies of DNA damage-induced HR

In the FYDR mice, HR-induced misalignments between two copies of an expression cassette for EYFP are detectable as fluorescent foci within intact pancreatic tissue (Fig. 1A,B). In some cases, foci are comprised of more than one fluorescent recombinant cell, indicative of a recombination event in a single cell that has subsequently undergone clonal expansion (Fig. 1C) [51]. Analysis of tissue histology shows that in the pancreas, acinar cells undergo HR (Fig. 1D), and previous studies show that acinar cells comprise virtually all of the recombinant cells in the FYDR pancreas [51].

The FYDR mice enable studies of exposure-induced HR in the pancreas of adult animals. Of particular interest are alkylating agents, an important class of DNA damaging agents that are present in food and in our environment, some of which have been shown to cause cancer [52–54]. Ironically, alkylating agents are used to treat cancer when given at high doses [55]. Temozolomide, a methylating agent that is used in cancer chemotherapy, kills tumor cells by creating DNA lesions that either directly or indirectly inhibit DNA replication, causing cytotoxicity [55]. Cells that do not die from exposure to temozolomide potentially run the risk of harboring chemotherapy-induced mutations, including HR events. To determine if alkylation damage induces HR in the pancreas, FYDR mice were exposed to the model methylating agent MNU, which creates the same types of base lesions as temozolomide. Results show that MNU causes a significant increase in the frequency of fluorescent foci (Fig. 1E), indicating that the FYDR mouse model is effective for studies of DNA damage-induced HR.

### Pancreatic inflammation induced by cerulein leads to edema and precancerous lesions

**Figure 2:**
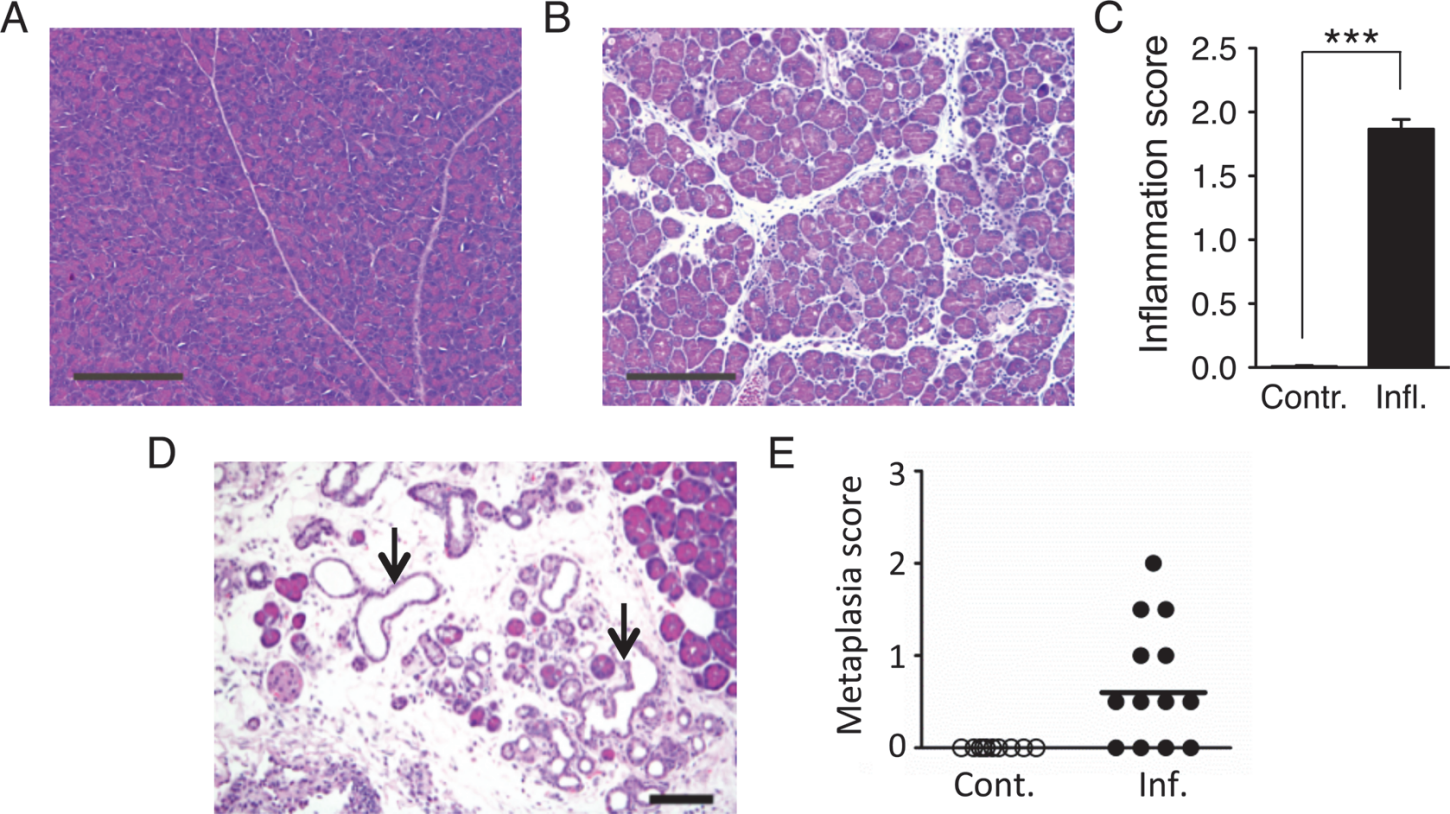
Cerulein treatment induces inflammation in the pancreas, and chronic cerulein pancreatitis induces metaplastic changes. (**A**) Tissue sections from pancreata of control mice show normal pancreas architecture. (**B**) Acute cerulein treatment induces pancreatic inflammation evidenced by edema and an inflammatory infiltrate. (**C**) Severity of cerulein-induced inflammation as determined by a trained pathologist. Inflammation scores are significantly higher in cerulein-treated mice (n = 30) than in control mice (n = 30). Data are mean ± SEM. *** *P* < 0.001 (Student’s *t*-test). (**D**) Pancreas section from a mouse treated with cerulein for 6 months shows chronic pancreatic inflammation, edema, significant acinar loss, and acinar to ductal metaplasia (arrows). (**E**) Quantification of metaplastic changes determined by a trained pathologist shows absence of metaplasia in control mice. However, 9 out of 13 mice treated with cerulein for 6 months show metaplastic changes. See *Methods* for detailed pathological scoring criteria. Statistical testing could not be performed in groups containing only zero values. Panels **A,B**: Original magnification, ×10. Scale bar = 200 μm. Panel **D**: Original magnification, ×200. Scale bar = 80 μm.

In order to study the interactions between DNA damage and inflammation, we exploited cerulein, a cholecystokinin analog that is well established as an inducer of pancreatic inflammation [56,57]. Animals exposed to cerulein by 6 hourly intraperitoneal injections showed pancreatic edema and infiltration by inflammatory cells, chiefly neutrophils (Fig. 2A,B). The extent of features of pancreatitis was found to be statistically significantly increased when quantified by a trained pathologist (Fig. 2C). In studies of long term exposure to cerulein, we observed severe tissue atrophy and metaplasia in wild type mice (Fig. 2D,E), and precancerous lesions in K-Ras mice (S2 Fig.), indicating that cerulein exposure serves as a relevant model for pancreatitis-induced cancer.

### Acute inflammation induces DSBs

**Figure 3:**
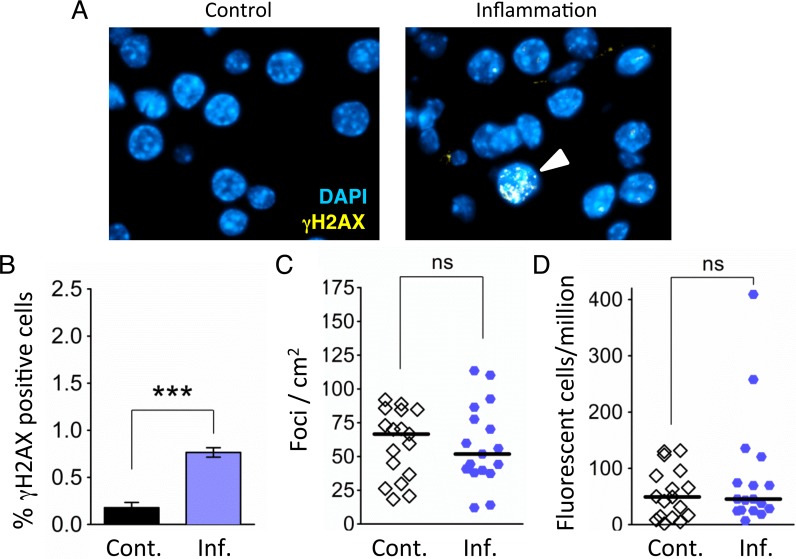
Independent bouts of inflammation induce DSB formation but not HR. (**A**) Immunohistochemical staining for the DSB marker γH2AX (yellow) in pancreas sections. Nuclei were counterstained with DAPI (blue). In control mice, nuclei with γH2AX foci are very rare (*Left*). However, nuclei with γH2AX foci (arrowhead) appear after independent bouts of inflammation (*Right*). (**B**) Quantification of nuclei containing more than five γH2AX foci shows significantly more γH2AX positive nuclei after inflammation (n = 6) than in control animals (n = 6). Data are mean ± SEM. *** *P* < 0.001 (Student’s *t*-test). (**C**) Numbers of fluorescent foci in the pancreas are not different between control mice (n = 17) and mice that underwent repeated acute inflammation (n = 17). Symbols represent data from individual mice, horizontal bars show medians. ns, not statistically significant (Mann–Whitney *U*-test). (**D**) No statistically significant difference in the frequencies of fluorescent cells in the pancreas between control mice (n = 17) and mice that underwent repeated acute inflammation (n = 17). Pancreata were disaggregated into single-cell suspensions and the frequencies of fluorescent cells were determined by flow cytometry. Symbols represent data from individual mice, horizontal bars show median values. ns, not statistically significant (Mann–Whitney *U*-test).

During inflammation, an increase in the levels of macrophages and neutrophils leads to increased levels of RONS [18]. RONS in turn induce base lesions including eA, 8oxoG and Hx, which have been observed at sites of inflammation [18,19]. Many RONS-induced DNA lesions have the potential to cause recombinogenic DSBs through chemical cleavage, by enzymatic processing, or as a result of replication fork breakdown [58–60]. To learn if pancreatic inflammation induces DSBs *in vivo*, we analyzed the frequency of DSB repair foci by quantifying cells with five or more γH2AX foci (H2AX becomes phosphorylated to form γH2AX in the vicinity of DSBs) [61]. Immunohistochemical (IHC) analysis of pancreatic tissue reveals a clear induction of DSBs after exposure to cerulein (Fig. 3A,B).

### Repeated exposure to acute inflammation does not cause a detectable increase in HR

As HR has been shown to be induced by DSBs *in vitro* [62,63], we next asked if DSBs associated with acute inflammation induce HR *in vivo*. To increase the sensitivity of our approach, animals were exposed to three bouts of acute pancreatitis. Analysis of the frequency of HR events in control animals shows that there is variation in the frequencies of foci/cm^2^, ranging from ∼15 to ∼100 (Fig. 3C), consistent with previous studies [7,64,65]. (It is noteworthy that variation in mutation frequency among normal animals has similarly been shown in several other mouse models for mutation detection [66–69]). Unexpectedly, in animals that were subjected to three bouts of inflammation, we did not detect any increase in the frequency of recombination events (indicated by fluorescent foci; Fig. 3C). Analysis of the frequency of fluorescent recombinant cells similarly did not reveal any increase in HR in the animals exposed to three bouts of inflammation (Fig. 3D).

### Inflammation-induced cell proliferation occurs days after infiltration and edema

**Figure 4:**
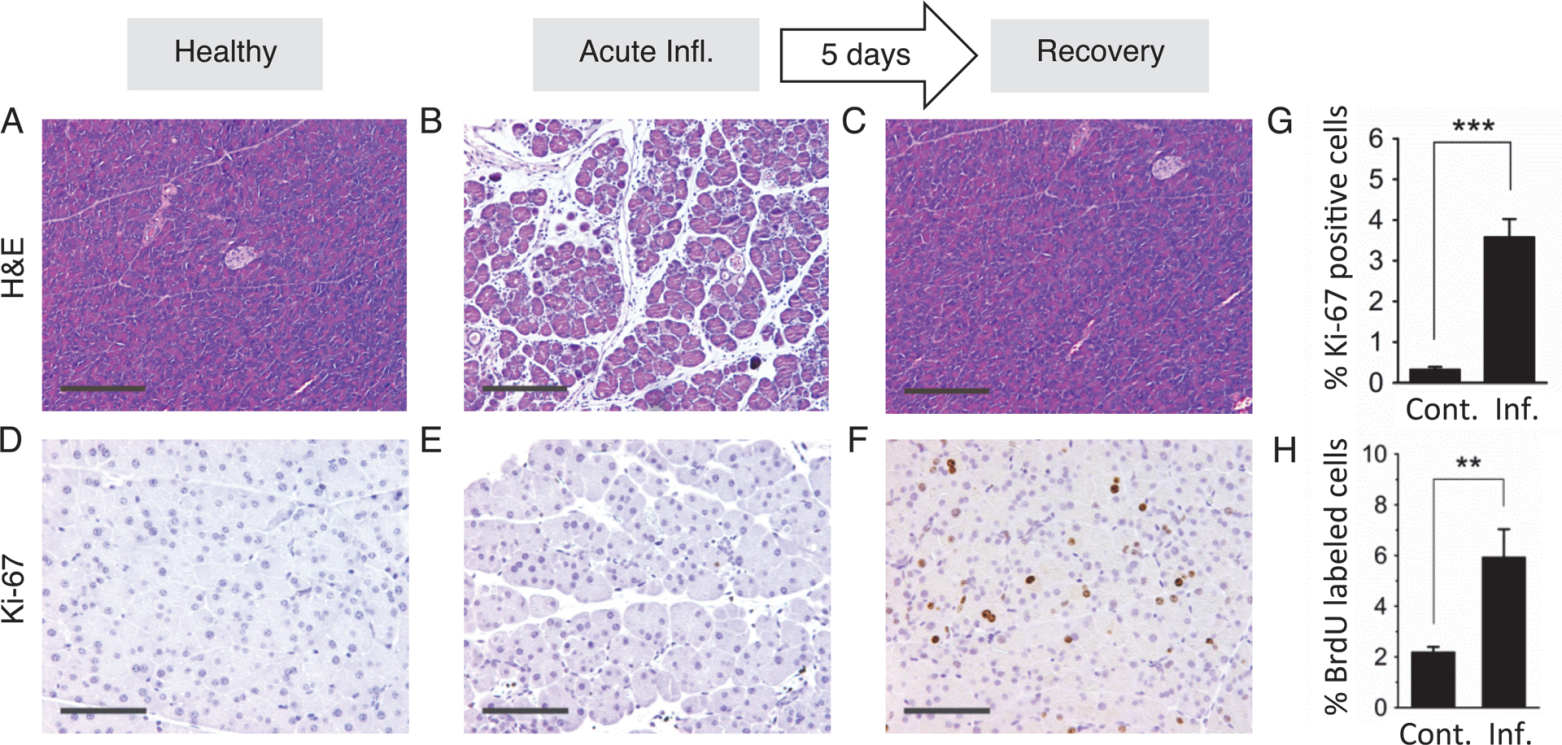
Inflammation and regenerative cell proliferation are separated in acute cerulein pancreatitis. (**A**) Pancreas from control mouse showing normal tissue architecture with no detectable histological changes. (**B**) 12 hours after acute cerulein treatment, the pancreas shows histological signs of acute pancreatitis, such as edema and an inflammatory infiltrate. (**C**) Five days after acute cerulein treatment, inflammation is no longer detected and histology is comparable to healthy tissue. (**D**) Low Ki-67 staining indicates low proliferative activity in control pancreata. (**E**) Ki-67 staining remains low during acute pancreatitis, indicating no increase in cell proliferation during acute inflammation. (**F**) Five days after acute cerulein treatment, increased Ki-67 staining indicates increased cell proliferation during tissue regeneration. (**G**) Quantification of Ki-67 labeling shows significantly higher proliferation in regenerating tissue. Data are mean ± SEM in control mice (n = 16) and in mice with acute pancreatitis (n = 16). *** *P* < 0.001, Student’s *t*-test. (**H**) Increased cell proliferation during regeneration from acute pancreatitis is indicated by increased BrdU labeling. Five days after acute pancreatitis or mock treatment, mice received BrdU (75 mg/kg i.p.) to label newly replicated DNA in proliferating cells. Pancreata were harvested 4 hours later, disaggregated, and the frequencies of BrdU labeled cells were determined by antibody staining and flow cytometry. Data are mean ± SEM in control mice (n = 5) and in mice with acute pancreatitis (n = 5). ** *P* < 0.01, Student’s *t*-test. Panels **B,C,D**: Original magnification, ×10. Scale bar = 200 μm. Panels **E,F,G**: Original magnification, ×20. Scale bar = 100 μm.

HR is active during S/G_2_, whereas most cells in healthy pancreatic tissue are non-dividing cells in G_0_/G_1_ [70], raising the possibility that HR was not active in RONS-exposed cells during the three bouts of inflammation. To learn about the extent of cell division during the course of inflammation, we quantified dividing cells when tissue is healthy (Fig. 4A), subject to acute inflammation (Fig. 4B) or recovering (Fig. 4C; five days after cerulein exposure, when features of inflammation have cleared). Cell proliferation during the course of the inflammatory response was evaluated by staining for Ki-67, a marker of cell proliferation [71]. Results show that there are very few Ki-67 positive cells in control and acutely inflamed tissue (Fig. 4D,E). In contrast, the frequency of Ki-67 positive cells is significantly induced during tissue recovery (Fig. 4F) and when quantified using image analysis software (see Materials and Methods) (Fig. 4G). As an alternative approach, animals were treated with BrdU, a thymidine analog that becomes integrated into the DNA of dividing cells and can be detected using immunohistochemistry. Pancreatic tissue was disaggregated, and the frequency of BrdU positive cells was analyzed by flow cytometry. Consistent with the Ki-67 analysis, results show a clear increase in the frequency of dividing cells several days after acute inflammation (Fig. 4H). Thus, with both methods, we found that acute phase inflammation is separate from a subsequent proliferative phase.

### Creating overlap between the acute and proliferative phases of inflammation causes sequence rearrangements

**Figure 5:**
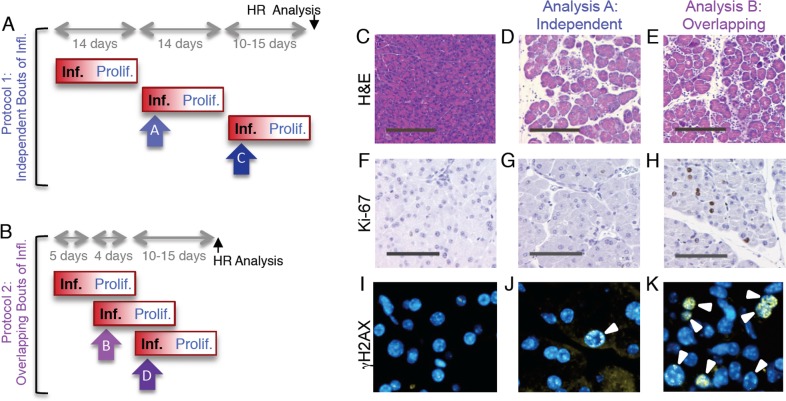
Independent and overlapping bouts of pancreatic inflammation. (**A**) For independent bouts of inflammation, three acute cerulein pancreatitis events were induced two weeks apart, and inflammation and proliferation were assessed at the second (analysis time A) and third (analysis time C) bout of inflammation. HR was quantified 10 to 15 days after the last pancreatitis event. (**B**) For overlapping bouts of inflammation, three acute cerulein pancreatitis events were induced on days 1, 4 and 9. Inflammation and proliferation were assessed at the second (analysis time B) and third (analysis time D) bout of inflammation. HR was quantified 10 to 15 days after the last pancreatitis event. (**C**) Pancreas section from a control mouse shows healthy tissue. (**D,E**) Treatment with cerulein (both independent and overlapping) results in edema and an inflammatory infiltrate chiefly of neutrophils, indicating acute inflammation. (**F**) Ki-67 immunohistochemistry shows low levels of baseline proliferation in control pancreata. (**G**) Cell proliferation remains low in the pancreas during acute inflammation. (**H**) During regeneration from acute inflammation, Ki-67 positive nuclei appear, indicating regenerative proliferation. (**I**) Immunohistochemical detection of γH2AX phosphorylation in pancreas sections show low levels of DSBs in healthy pancreata. (**J**) During independent bouts of inflammation, nuclei with γH2AX foci (arrowhead) become apparent. (**K**) During overlapping bouts of inflammation, more γH2AX positive nuclei are visible. (**C**-**E**) Original magnification, ×10. Scale bar = 200 μm. (**F**-**H**) Original magnification, ×20. Scale bar = 100 μm. (**I**-**K**) Original magnification, ×40.

As HR is active primarily during S/G_2_, we hypothesized that the lack of HR induction following three independent bouts of inflammation might be due to the kinetic separation between acute inflammation-induced DSBs and recovery-induced cell proliferation. We therefore asked if inflammation might induce HR if the timing were adjusted to create overlap between inflammation-induced DSBs and cell proliferation. For ‘protocol 1’ described above, animals were exposed to three independent bouts of inflammation, each two weeks apart (Fig. 5A). Here, for ‘protocol 2’, animals were also exposed to three bouts of inflammation, however bouts of inflammation were 4–5 days apart (Fig. 5B).

For ‘protocol 1’, we observed that exposure to cerulein induces acute inflammation, as can be seen by the edema and infiltration under inflamed conditions (compare Fig. 5C and 5D). At the time of acute inflammation, the frequency of dividing cells is unchanged compared to untreated animals (Fig. 5F,G). However, cells with high numbers of γH2AX foci are apparent (Fig. 5J), which is consistent with DNA damage formed by RONS that are associated with the acute phase of inflammation. We also observed acute inflammation using ‘protocol 2’ (Fig. 5E). Unlike protocol 1, we also observed concomitant induction of cell division, consistent with the proliferative phase of the first bout of inflammation (Fig. 5H). Cells with high frequencies of γH2AX foci are evident (Fig. 5K).

**Figure 6:**
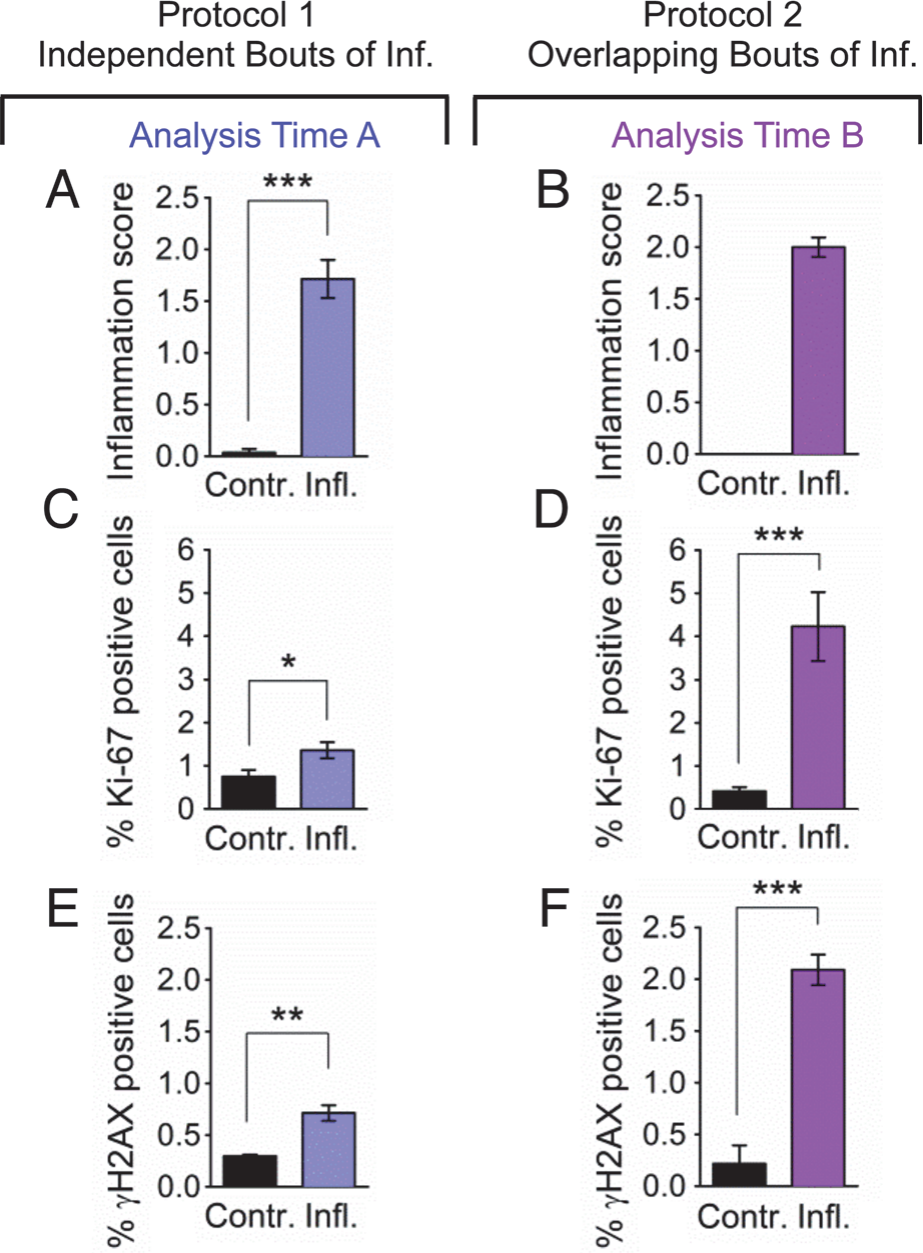
Overlapping bouts of inflammation induce more DSBs than independent bouts of inflammation. Inflammation, cell proliferation and γH2AX foci formation were quantified in pancreas sections from mice treated with independent bouts of inflammation (blue bars) and with overlapping bouts of inflammation (purple bars). (**A,B**) Cerulein induces inflammation in both independent (n = 7) and overlapping (n = 8) treatment regimens. Severity of inflammation in control and cerulein-treated mice was quantified by a trained pathologist. (**C, D**) Quantification of nuclei positive for the proliferation marker Ki-67 shows a moderate increase in independent bouts of inflammation (n = 7), and a large increase in overlapping bouts of inflammation (n = 8). (**E,F**) Quantification of nuclei positive for the DSB marker γH2AX (nuclei with >5 foci) shows a moderate increase in independent bouts of inflammation (n = 3), and a large increase in overlapping bouts of inflammation (n = 3). Data are mean ± SEM. See *Methods* for detailed pathological scoring criteria. Statistical testing could not be performed in groups containing only zero values. * *P* < 0.05; ** *P* < 0.01, *** *P* < 0.001 (Student’s *t*-test).

To learn more about the impact of overlap between bouts of inflammation, the extent of inflammation was assessed by a trained pathologist, the extent of cell proliferation was quantified by automated image analysis, and the frequency of γH2AX positive cells was measured by counting cells with >5 γH2AX foci. Results show that the severity of the acute phase of inflammation is similar regardless of whether bouts of inflammation occur independently or in an overlapping fashion (Fig. 6A,B). In contrast, cell proliferation is dramatically increased under conditions where the response to the first bout of inflammation overlaps with initiation of the second bout of inflammation (Fig. 6C,D). The frequency of DSBs is increased in both independent and overlapping bouts of inflammation, and the increase is greater under conditions of overlap between the acute phase of inflammation and the proliferative phase (compare Fig. 6E and Fig. 6F). Similar results were observed for the third bout of inflammation (S3–S4 Fig.), although the frequencies of γH2AX were reduced during the third bout of inflammation relative to the second bout under conditions of overlap. It is unclear why the third bout of inflammation is apparently less damaging, however one possibility is that HR proficiency increased during the course of the exposure protocol, leading to more rapid clearance of DSBs. It is noteworthy that clearance of potentially toxic DSBs is advantageous to cell survival, but carries the risk of mutations due to HR misalignments.

**Figure 7:**
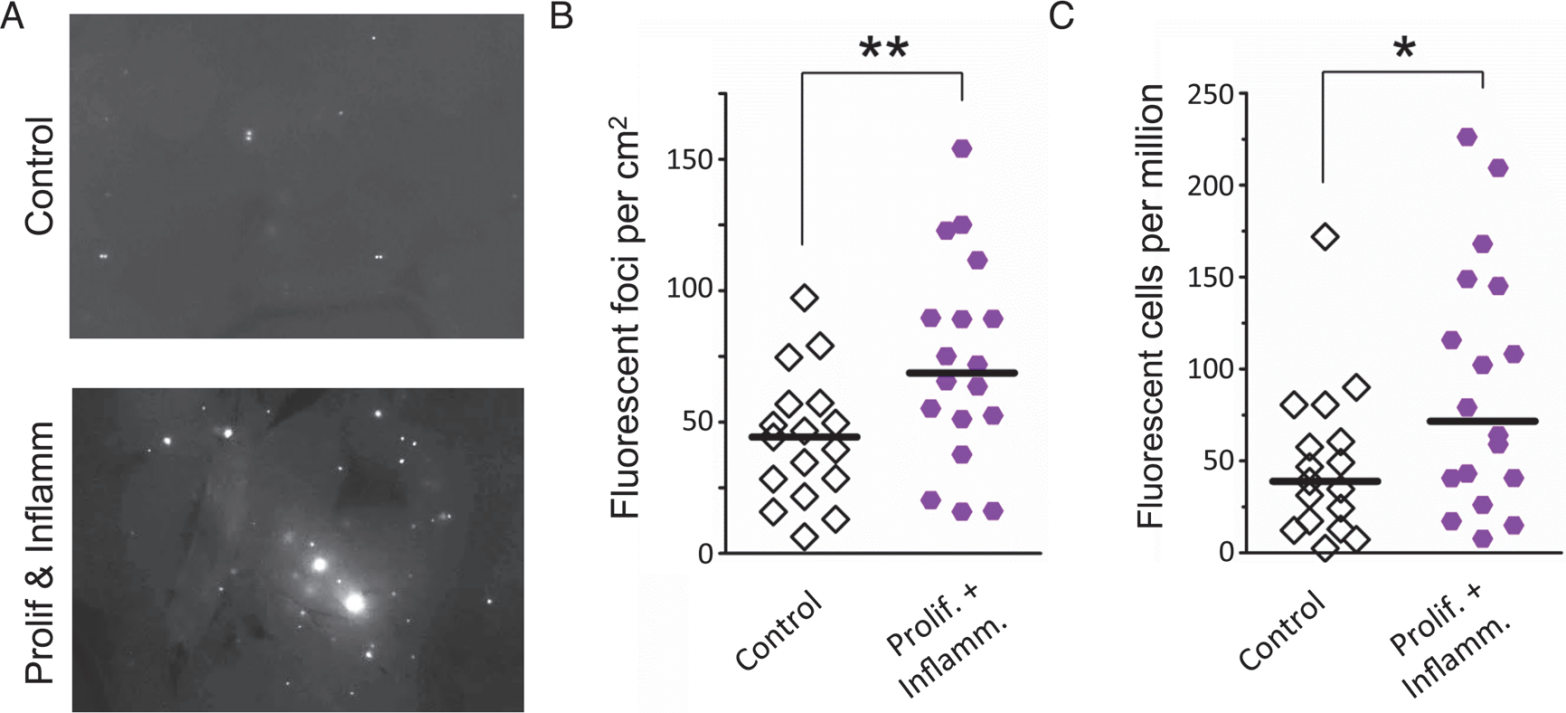
Simultaneous inflammation and cell proliferation induces HR in the pancreas. (**A**) Representative images from pancreata of control mice (*Top*) and mice that experienced combined proliferation and inflammation (*Bottom*). Freshly harvested whole organs were compressed between glass coverslips and imaged under an epifluorescent microscope. Representative details of composite images are shown, fluorescent foci are apparent *in situ*. More foci are visible in the pancreas from the proliferation plus inflammation group. Brightness and contrast have been enhanced identically. (**B**) Numbers of fluorescent foci are higher in mice that experienced combined proliferation and inflammation (n = 18) than in control mice (n = 17). Symbols represent data from individual mice, horizontal bars show medians. **, *P* < 0.01, (Mann–Whitney *U*-test). (**C**) Higher fluorescent cell frequency in the pancreata of mice that experienced combined proliferation and inflammation (n = 18) than in control mice (n = 17). Pancreata were disaggregated into single-cell suspensions and the frequencies of fluorescent cells were determined by flow cytometry. Symbols represent data from individual mice, horizontal bars show median values. *, *P* < 0.05 (Mann–Whitney *U*-test).

To learn about the impact of inflammatory response kinetics on susceptibility to HR, recombination events were quantified within intact pancreatic tissue, and the frequency of recombinant cells was evaluated in disaggregated pancreatic tissue by flow cytometry. Under conditions of overlapping bouts of inflammation (protocol 2), there is a significant increase in the frequency of recombination events, which is both visually apparent (Fig. 7A) and quantitatively significant (Fig. 7B). In addition, there is a significant increase in the frequency of fluorescent recombinant cells under conditions of overlap (Fig. 7C), but not when animals are exposed to three independent bouts of inflammation (Fig. 3D).

### Inflammation potentiates rearrangements induced by a model cancer chemotherapeutic

The observation that overlapping bouts of inflammation induce HR is consistent with a model wherein inflammation-induced cell proliferation sensitizes tissue to HR induced by endogenously-produced DNA damage. We next asked about the potential for inflammation-induced cell proliferation to cause increased sensitivity to HR induced by an exogenous DNA damaging agent, specifically the model cancer chemotherapeutic, MNU.

**Figure 8:**
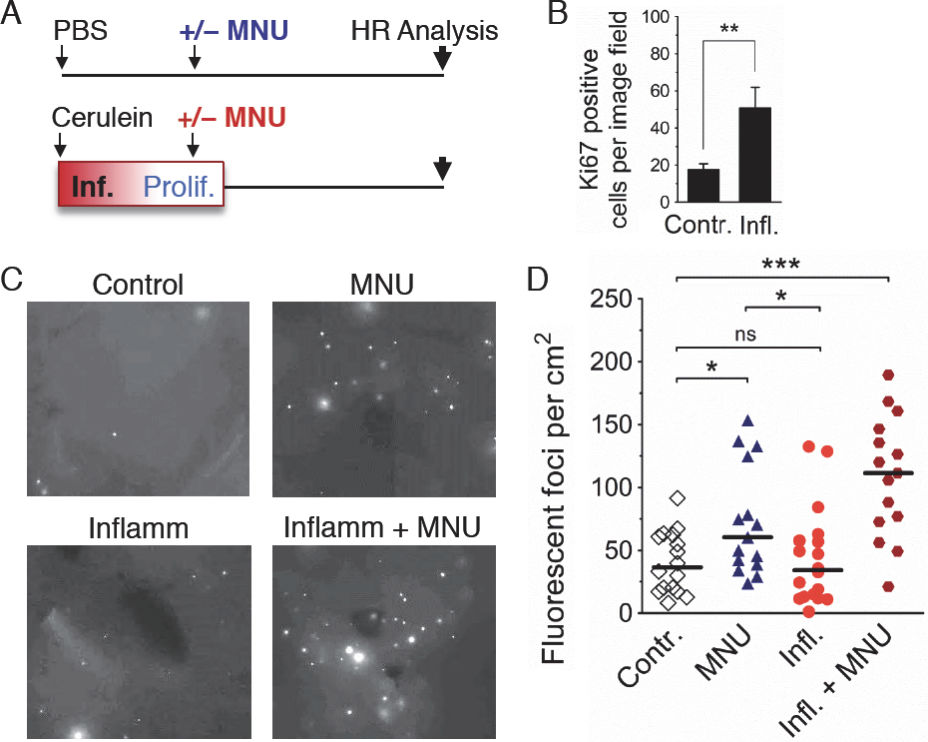
Inflammation-associated cell proliferation potentiates the effect of exogenous DNA damage on DNA rearrangements. (**A**) Treatment scheme. Mice were subjected to a single acute cerulein pancreatitis event or mock treatment. At the peak of replacement proliferation, mice received MNU (25 mg/kg i.p.) or mock treatment. 3 to 4 weeks after MNU injection, mice were humanely sacrificed for HR analysis. (**B**) Replacement proliferation in the pancreas is indicated by increased Ki-67 expression. Five days after acute pancreatitis or mock treatment, pancreata were harvested and processed for Ki-67 immunohistochemistry. Data are mean ± SEM in control mice (n = 7) and in mice with acute pancreatitis (n = 8). ** *P* < 0.01, Student’s *t*-test. (**C**) Representative images from pancreata after inflammation and/or exogenous DNA damage. Freshly harvested whole organs were compressed between glass coverslips and imaged under an epifluorescent microscope. Representative details of composite images are shown, fluorescent foci are apparent *in situ*. More foci are visible after treatment with MNU, and a large increase is evident after treatment with MNU during regenerative proliferation (Inflamm+MNU panel). (**D**) Quantification of fluorescent foci in pancreata after inflammation and/or exogenous DNA damage. The number of fluorescent foci is significantly higher in MNU-treated mice (n = 15) than in control mice (n = 16), but there is no statistically significant increase after a single acute inflammation event (n = 18). However, there is a large increase in the number of foci after treatment with MNU during regenerative proliferation (Inflamm + MNU, n = 15). Symbols represent data from individual mice, horizontal bars show median values in each group. *, *P* < 0.05; ***, *P* < 0.001 (Mann–Whitney *U*-test).

Experiments were designed with the objective of finding the time when inflammation-induced cell proliferation is high, and then exposing animals to MNU (Fig. 8A). To quantify the extent of inflammation-induced proliferation, pancreatic tissue was analyzed for Ki-67 positive cells. There is a significant increase in cell proliferation at the time of the MNU exposure (Fig. 8B). MNU on its own causes a visually apparent (Fig. 8C) and statistically significant increase in the frequency of HR events in healthy animals (Fig. 8D) (note that the data from Fig. 1E have been replotted to facilitate comparisons among cohorts). The effect of MNU on HR was dose dependent: at 25 mg/kg, there was a statistically significant increase in the number of fluorescent foci (Fig. 8D), whereas there was not a significant increase in HR after treatment with 7.5 mg/kg MNU (S5 Fig.). We also found that a single bout of inflammation does not induce HR (Fig. 8C,D), which is consistent with results shown above (Fig. 3). Importantly, when animals were exposed to MNU at a time when inflammation-induced proliferation is high, there was a dramatic increase in the frequency of HR (Fig. 8C,D), revealing that physiological changes associated with inflammation and exposure to an exogenous DNA damaging agent act synergistically to induce HR. These results call attention to the importance of inflammation as a modulator of DNA damage-induced sequence rearrangements induced by exposure to an alkylating agent that serves as a model for environmental and clinical DNA damaging agents.

## Discussion

**Figure 9:**
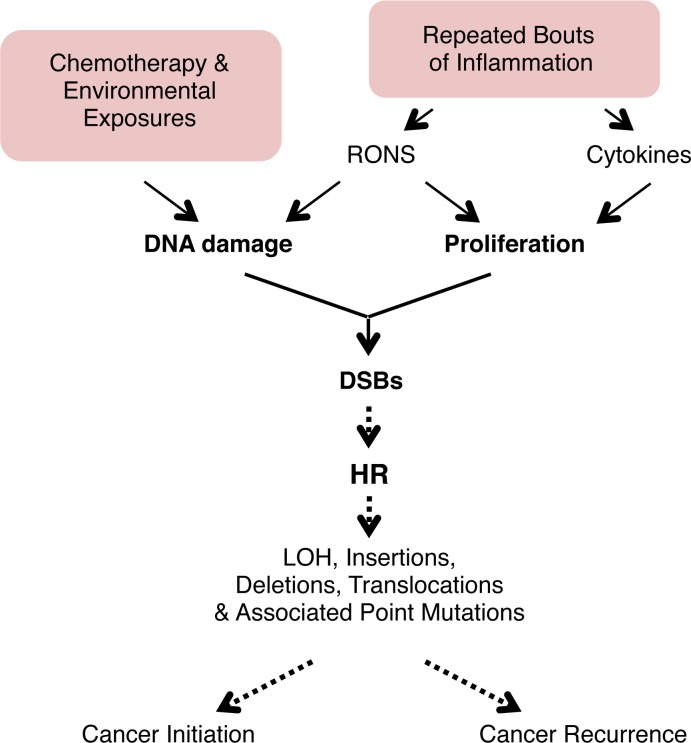
Model for the potentiation of sequence rearrangements induced by endogenous and exogenous DNA damage by inflammation-associated cell proliferation. Cell proliferation associated with inflammation may be induced by RONS released from inflammatory cells. Regeneration after inflammation also involves cell proliferation to replenish cells lost to inflammation-induced tissue damage. DNA replication is increased in proliferation, and DNA damage during replication can lead to fork breakdown and the formation of DSBs. These DSBs are repaired by HR, but HR can result in LOH, sequence rearrangements, and point mutations. Thus, cell proliferation potentiates the deleterious effect of both endogenous (RONS-induced) and exogenous (exposure-induced) DNA damage, potentially contributing to cancer initiation and recurrence. See text for details.

Pancreatic cancer is one of the most deadly cancers, yet relatively few studies have explored factors that govern susceptibility to mutations that initiate pancreatic cancer. Furthermore, while radiation and chemotherapy can be effective initially, recurrence is virtually inevitable [72], and mutations are a key driver of recurrence since they enable evolution into drug resistant and more aggressive phenotypes [12–15]. Thus, there is a need for a deeper understanding of the mechanisms of DNA damage-induced mutations in the pancreas. Furthermore, while it is well established that pancreatitis is a key risk factor for pancreatic cancer [11,16], studies had not previously been done to explore how physiological changes associated with inflammation modulate the risk of mutations *in vivo*. Here, we show that pancreatic inflammation leads to DNA double strand breaks, and that pancreatitis is associated with hyperproliferation. By creating conditions where there is overlap between bouts of inflammation, we show that DSBs and hyperproliferation act synergistically to induce sequence rearrangements *in vivo* (Fig. 9), which both demonstrates a correlation between DSBs and HR *in vivo* and provides insights into the underlying mechanisms that make pancreatitis a risk factor for cancer. Furthermore, we show that inflammation-induced proliferation acts synergistically with a DNA alkylating agent to induce sequence rearrangements *in vivo*, providing new understanding into factors that modulate the risk of sequence changes that promote cancer.

For decades, it has been known that inflammation is a risk factor for cancer [11,16], and it has long been postulated that it is the combination of inflammation-induced DNA damage and inflammation-induced cell proliferation that plays a key role in promoting mutagenesis [8–11]. Nevertheless, direct evidence for this model was lacking. Here, we show that, unexpectedly, several bouts of acute inflammation on their own are not sufficient to drive sequence rearrangements, and that separation of the acute phase of inflammation (associated with RONS and DNA damage) and the proliferative stage of inflammation provides a barrier to DNA damage-induced sequence rearrangements. Consequently, conditions that lead to chronic inflammation may be more likely to potentiate tumorigenic mutations compared to isolated bouts of inflammation, which is consistent with epidemiological studies [73,74].

Here, we observed that approximately half of the animals exposed to overlapping bouts of inflammation have frequencies of recombinant cells that are ∼100–200% higher than the untreated control animals. Given that the mutation rate can be rate limiting in tumor promotion [14], a doubling of the mutation frequency could potentially double the probability of cancer recurrence. An increased risk of mutations has relevance to many medical conditions that are associated with chronic inflammation [4]. Inflammatory bowel diseases such as ulcerative colitis and Crohn’s disease involve chronic inflammation in the colon, while chronic esophagitis and pancreatitis affect the upper gastrointestinal tract and the pancreas respectively. In addition, chronic infections with bacteria, viruses and parasites can lead to chronic inflammation at multiple sites. Importantly, chronic inflammatory conditions typically last for an extended period of time. Thus, a relatively small increase in susceptibility to mutations in people is anticipated to become very significant given the accumulation over a period of years.

RONS create a wide array of DNA lesions that includes dozens of different types of base lesions as well as abasic sites and strand breaks [75–77]. There is a wealth of information about the mutagenicity of RONS-induced DNA damage derived from studies in vitro [78,79]. Many elegant studies have revealed the mutagenic potential of specific RONS-induced base lesions using site-specific lesion technology [80], and many others have described the ability of inflammatory chemicals to induce mutations in RONS-exposed cells in vitro [81,82]. Using these and other approaches, we now know quite a lot about the molecular and biochemical mechanisms of RONS-induced mutagenesis. For example, 8oxoG readily mispairs with thymine when bypassed by translesion polymerases [83,84], and that cells prevent TLS-driven mutagenesis by removing 8oxoG [85–87]. Cells also have additional strategies for preventing RONS-induced mutations, including removal of damaged bases from the nucleotide pool (e.g., Mth1) [88,89], and removing the misincorporated base opposite the lesion post replication (e.g., removal of adenine across from 8oxoG by Mutyh) [89, 90]. While the literature describing RONS-induced base lesions *in vitro* is extensive (we refer the reader to several excellent reviews [78,79,81,82]), relatively few studies have addressed RONS-induced mutagenesis *in vivo*. These studies showed that base excision repair is critical in suppressing RONS-induced mutations *in vivo* [91–94], and that inflammation induces mutations in the affected tissues [95–97]. Interestingly, in one such study it was shown that *H. pylori* infection is associated with mutations [98], however the frequency of mutations decreased when Ogg1 was knocked out, leaving unclear the mechanism of mutagenesis. In another study, Ogg1 was found to suppress mutations induced by oxidative damage [99]. The most direct evaluation of the relationships among inflammation, DNA damage, mutagenesis, and cancer was done recently in the laboratory of L. Samson. This study showed that a deficiency in the Aag glycosylase is associated with increased inflammation-induced cancer, and that tumors harbor mutations consistent with the predicted mutations that would result from an Aag deficiency [6].

Here, we have extended the *in vivo* studies of inflammation and mutagenesis to specifically query the inter-relationships among inflammation, cell proliferation, DSBs, and their consequences (homologous recombination events), using tools that had not previously been applied to this problem. It is important to note that this study focuses on a specific class of mutation (HR-driven sequence rearrangements), and that there are other classes of mutations that are not detected by the FYDR assay, such as base damage-induced point mutations (which often arise during TLS), and small insertions/deletions (which are sometimes associated with NHEJ). Nevertheless, inflammation-induced HR events are expected to arise contemporaneously with other classes of mutations. Specifically, both point mutations and HR events arise primarily as a consequence of DNA damage that is present during DNA replication. Thus, HR may serve as an indicator of a more general increase in mutagenesis. Indeed, an association between point mutations and HR events is consistent with observations showing that exposure-induced HR is an excellent predictor of carcinogenicity, which generally arises as the result of multiple classes of mutations [100].

RONS-induced DSBs are rarely caused by direct reaction with the DNA [18,19], but instead are the result of enzymatic processing. Specifically, base excision repair of RONS-induced lesions is associated with gaps that form as repair intermediates [60]. These single strand breaks can become DSBs when repair patches are closely opposed [60,101]. Additionally, replication forks that encounter RONS-induced single strand breaks can break down [21], creating a double strand break. We observed an increase in DSBs under both the conditions of isolated bouts of inflammation, and overlapping bouts of inflammation. Interestingly, under conditions where proliferation from the first bout of inflammation overlaps with acute inflammation from the second bout of inflammation, we observed that DSBs were greatly increased compared to conditions without overlap. This observation is consistent with the possibility that DSBs form in a replication-dependent manner as a result of replication fork breakdown.

In the FYDR direct repeat substrate, full-length *Eyfp* sequence can be reconstituted by several HR mechanisms. For example, if there is a fork breakdown event during DNA replication, misinsertion of the double-strand end can restore full length *Eyfp*, leading to a gain of one repeat unit (a rearrangement at the FYDR substrate, Fig. 1A). Importantly, the FYDR substrate is similar in size to *Alu* repeats (∼500 bp vs ∼300 bp), which make up almost 10% of the human genome and are frequent sites of HR-induced rearrangement formation [102]. HR between *Alu* repeats can yield deletions, duplications and translocations [102]. *Alu*-mediated rearrangements have been shown to activate oncogenes in cancer [103] and to inactivate tumor suppressor genes such as p53 [104]. Further, HR-driven rearrangements between *Alu* repeats have been shown to drive carcinogenesis in inflammation-associated cancers [36, 105]. Thus, HR events that occur in FYDR mice after replication fork repair are related to genetic changes that are relevant for carcinogenesis in humans.

Alkylating agents are abundant in our environment, endogenously produced in our cells, and used at high doses as cancer therapeutics. Understanding factors that modulate alkylation-induced mutations is therefore relevant both to cancer etiology and to cancer recurrence. We show here that inflammation-induced cell proliferation acts synergistically with alkylation damage to induce sequence rearrangements (Fig. 9). Thus, one potential factor when considering the underlying mechanisms by which chronic inflammation promotes cancer is that the inflammatory response sensitizes tissue to exposure to DNA damaging agents that are in our environment and in our food. Furthermore, as proliferation itself is sufficient to increase susceptibility to DNA damage induced sequence rearrangements [106], careful consideration should be given to babies *in utero* and young children for whom high levels of cell proliferation are anticipated to greatly sensitize cells to exposure-induced mutations. Thus, when screening for potentially carcinogenic exposures, it will be important to consider the importance of a person’s physiological state when assessing risk, with regard to both chronic inflammatory conditions and stage in development.

Recurrence is the single biggest hurdle in cancer treatment, and mutations are critical in eliciting phenotypic changes that initiate new secondary cancers, promote existing cancer cells, and potentiate drug resistance [1,12–15]. It has recently been demonstrated that mutation rate directly impacts the emergence of drug resistance [14]. While in some cases cancer cells are hypermutable [13], many transformed cells have a normal mutation rate [12], making exposure-induced mutations highly relevant. Tumors generally exist in a chronic pro-inflammatory environment. Associated increases in proliferation of both tumor and stromal cells are anticipated to increase susceptibility to RONS-induced and chemotherapy-induced HR events that can promote metastasis and recurrence (Fig. 9). Novel approaches for treating cancer are currently in development, including staged release of drugs from nanoparticles that increase cell killing by chemotherapeutic agents [107]. These approaches could help minimize treatment-induced mutations and thus slow the emergence of drug resistant or more aggressive cancers.

The observation that there is synergy between conditions that induce hyperproliferation and conditions that cause DNA damage is relevant to millions of people who suffer from chronic inflammation and are thus at increased risk of mutations that drive cancer. In addition, the observation that inflammation sensitizes tissue to alkylation-induced HR is relevant to other exposures that create DNA lesions that inhibit replication, including constituents of food, cigarette smoke, and environmental carcinogens (*e.g*., aflatoxin, BaP, PhIP). Importantly, although the focus of this work is on HR at an integrated reporter, the FYDR model serves as a powerful tool to learn about more general increases in HR throughout the genome, with their accompanied increased risk of LOH, insertions, deletions, and point mutations, all of which drive cancer (Fig. 9). Through these studies of the dynamic physiological changes associated with inflammation, this work contributes to our fundamental understanding of how inflammation drives genetic changes that cause cancer and calls attention to new avenues to disease prevention and treatment.

## Materials and Methods

### Ethics statement

All animal experiments were conducted according to the Guide for the Care and Use of Laboratory Animals, and were approved by the MIT Committee on Animal Care.

### Chemicals

Cerulein, methylnitrosourea (MNU), BrdU, soybean trypsin inhibitor and collagenase were purchased from Sigma-Aldrich.

### Animals

Female C57Bl/6 *p* ^un^ FYDR mice ([7], 5 to 7 weeks old) were used for measuring HR. Inflammation, proliferation and double-strand breaks were measured using female C57Bl/6 (Taconic) and C57Bl/6 p^un^ FYDR mice (5 to 7 weeks old). Metaplastic and preneoplastic lesions were assayed using male wild type or K-Ras mutant mice (gifts from T. Jacks, MIT) on the FVB background (8 months old at analysis). Mice were housed in an AAALAC approved, specific pathogen free facility under a 12h light/dark cycle and were fed a standard rodent diet (LabDiet RMH 3000, Purina LabDiet) and autoclaved water *ad libitum*. For measuring HR, litters were split between experimental groups.

### Repeated acute pancreatitis

Mice were subjected to 3 episodes of acute pancreatitis on experimental days 0, 4 and 9, or on days 0, 14 and 28. Each episode was elicited by 6 hourly intraperitoneal injections of cerulein (dissolved in PBS, 50 μg/kg for each injection). Control animals did not receive injections, as serial injections of PBS have no effect on HR (S6 Fig.). To assess inflammation, Ki-67 expression, and double-strand breaks, mice were humanely euthanized 12 hours after the first cerulein injection and pancreata were harvested for histological analysis. To assess regenerative cell proliferation by BrdU labeling, mice were dosed with BrdU (75 mg/kg) five days after the first bout of acute pancreatitis. Four hours after BrdU injection, mice were humanely euthanized and their pancreata were harvested and processed for BrdU detection by flow cytometry. To assess homologous recombination, mice were humanely euthanized 10 to 15 days after the last pancreatitis episode, and pancreata were harvested for the FYDR assay.

### Chronic pancreatitis

Chronic pancreatic inflammation was elicited by cerulein injections (5 μg dissolved in saline, single intraperitoneal injection, 5 days a week) for 6 months, as described in [108]. Control mice received saline injections. Mice were 2 months old at the beginning of treatment. At 8 months of age, mice were humanely euthanized and pancreata were harvested for histological analysis.

### Regenerative proliferation and exogenous DNA damage

Mice received 6 hourly intraperitoneal injections of cerulein (dissolved in PBS, 50 μg/kg for each injection) to induce acute pancreatitis. Control mice received 6 hourly injections of PBS. To assess regenerative proliferation by Ki-67 expression, mice were humanely euthanized five days after acute pancreatitis induction and their pancreata were harvested for histological analysis. To induce exogenous DNA damage during regenerative proliferation, mice were dosed with methylnitrosourea (25 mg/kg, dissolved in PBS, pH 4) five days after cerulein treatment. (Note that the timing in this experiment is different from the timing in the repeated inflammation experiment, as MNU generates DNA damage directly and much faster than inflammation induced by cerulein.) Control mice were dosed with PBS, pH 4. Mice were humanely euthanized 3 to 4 weeks after methylnitrosourea injection and pancreata were harvested for the FYDR assay.

### BrdU labeling

Pancreata were disaggregated by mechanical chopping and collagenase V digestion at 37°C for 40 min, followed by gentle pipetting. Cells were collected by centrifugation and were stained with the APC Cell Proliferation Detection Kit (BD Pharmingen) according to the manufacturer’s instructions. Samples were analyzed on a FACSCalibur flow cytometer (BD Biosciences) using CellQuest Pro software. On average, 20 000 cells were analyzed per sample.

### Ki-67 immunohistochemistry

Pancreata were fixed in 10% neutral buffered formalin, embedded in paraffin, and sectioned at 4 μm. After deparaffinization, heat-induced antigen retrieval was performed using a modified citrate buffer (Dako). Ki-67 antibody (rat anti-mouse Ki-67, Dako) was used at a dilution of 1/100 at room temperature for 1 hour. Secondary antibody (biotinylated rabbit anti-rat Ig, Dako) was used at a dilution of 1/100 at room temperature for 20 minutes, and detected using streptavidin-conjugated peroxidase and DAB. Sections were counter-stained with hematoxylin. In repeated inflammation experiments, the percentage of Ki-67 positive nuclei was determined in 20 randomly selected images (×20) using image analysis software (Visiopharm, Hørsholm, Denmark). In the acute inflammation + MNU experiment, the number of Ki-67 positive nuclei was counted in 15 randomly selected image fields (×20) in a blinded fashion.

### γH2AX immunofluorescence

Sections (4 μm) of formalin-fixed, paraffin-embedded tissue were deparaffinized and antigen-retrieved using modified citrate buffer (Dako). Sections were incubated with primary γH2AX antibody (Millipore) at a dilution of 1/100 at 4°C for 3 hours. Secondary antibody (Alexa Fluor 488 Goat Anti-Mouse IgG, Invitrogen) was used at a dilution of 1/500 at room temperature for 1 hour. Sections were counter-stained with DAPI before imaging. For each section, images of 20 randomly selected image fields were acquired at a magnification of ×40 using ImagePro Plus software (Media Cybernetics). DAPI-stained nuclei were counted using ImagePro Plus, and nuclei containing more than 5 γH2AX foci were counted manually in a blinded fashion.

### Homologous recombination assay

***In situ* fluorescent imaging**. Pancreata were immersed in ice cold soybean trypsin inhibitor solution (0.01% in PBS) immediately after harvesting. Pancreata were pressed between glass coverslips separated by 0.5 mm spacers and imaged on a Nikon 80*i* epifluorescence microscope (Nikon) with a CCD camera (CoolSNAP EZ, Photometrics) using a ×1 objective at a fixed exposure time (2 s). EYFP was detected in the FITC channel. Multipoint images captured using an automated stage (ProScan II, Prior Scientific) and NIS Elements software (Nikon) were stitched automatically or manually in Adobe Photoshop (Adobe Systems). Brightness and contrast were adjusted identically across images, and foci were manually counted in a blinded fashion. Areas of pancreata were determined using ImageJ software (NIH) by manually tracing the pancreas outline.

**Flow cytometry**. Following imaging, pancreata were disaggregated into single-cell suspensions as described in [7], with minor modifications. Briefly, pancreata were minced with scalpel blades, followed by digestion with collagenase V (2 mg/ml in Hanks’ Balanced Salt Solution) for 40 min at 37°C. The resulting suspension was gently triturated to increase mechanical separation and filtered through a 70 μm cell strainer (BD Falcon) into an equal volume of media (DMEM F12 HAM with 20% FBS). Cells were collected by centrifugation, resuspended in 350 μl OptiMEM (Invitrogen) and filtered through 35 μm filter caps into flow cytometry tubes (Beckton Dickinson). Samples were analyzed on a FACScan cytometer (Beckton Dickinson) using CellQuest Pro software (Beckton Dickinson). On average, 1 800 000 cells were analyzed per sample.

### Pathological analysis

Pancreata were fixed in 10% buffered formalin, embedded in paraffin, sectioned (4 μm) and stained with hematoxylin and eosin. Pancreata were then examined and scored by a trained veterinary pathologist in a blinded fashion on a scale of 0 to 4 for the following individual features: inflammation, edema, hemorrhage, acinar degeneration/necrosis, acinar loss/atrophy, fat infiltration, fibrosis, acinar to ductal metaplasia (ADM), acinar/ductal hyperplasia, acinar dysplasia/neoplasia and ductal dysplasia/hyperplasia. For the acute studies, only a few relevant subsets were analyzed and scored, whereas for the chronic studies, the full set of criteria was assessed.

### Statistics

Inflammation, proliferation and double-strand break indices were compared with Student’s *t*-test. Numbers of recombinant foci, recombinant cell frequencies, and pathological scores do not follow a normal distribution and were compared with the Mann–Whitney *U*-test. All statistical analyses were performed in GraphPad Prism, Version 5.02 (GraphPad Software). A *P* value less than 0.05 was considered statistically significant.

## Supporting Information

S1 Fig HR at the FYDR recombination substrate is detected by fluorescence after gene conversion, sister chromatid exchange, and replication fork repair. Each expression cassette is missing different essential *EYFP* coding sequences, such that neither is able to express functional protein. Gene conversion can lead to the transfer of sequence information from one cassette to the other, restoring full-length *EYFP* coding sequence and giving rise to fluorescence. Each cassette can be the donor or the recipient in a gene conversion event. The entire HR reporter is copied during S phase, making it possible for crossovers between sister chromatids (gene conversion with crossover) to reconstitute full-length *EYFP*. Note that a long tract gene conversion event would be indistinguishable. HR repair of a broken replication fork can also be detected using the FYDR substrate. The breakdown of a replication fork moving from left to right is shown. Reinsertion of the broken Δ3*egfp* end into the Δ5*egfp* cassette can restore full length *EYFP. EYFP* can analogously be restored by repair of forks moving in the opposite direction (not shown). Single strand annealing initiated by a DSB between the repeated cassettes can be readily repaired, but these events will not reconstitute full-length EGFP and thus SSA cannot be detected.
(TIF)

S2 Fig Chronic cerulein treatment leads to dysplastic and preneoplastic changes in K-Ras mice. (**A**) Pancreas from mock treated K-Ras mutant mouse. Inflammation, acinar atrophy and interstitial fibrosis (arrow) are detectable. Acinar-to-ductal metaplasia is sparse. H&E staining. Original magnification, ×100. Scale bar = 160 μm. (**B**) Pancreas from K-Ras mutant mouse treated with chronic cerulein. Small focal proliferation of acinar tubules (thick arrow) with architectural and cytological atypia (dysplasia, low grade) surrounded by inflammation. Few acini with mucous metaplastic changes (thin arrow) are also present. Original magnification, ×400. Scale bar = 40 μm. (**C**) Histological scores for acinar-to-ductal metaplasia in mock and chronic cerulein treated K-Ras mutant mice. Detailed scoring criteria are described in *Methods*. Each symbol denotes data from one mouse. ***, *P* < 0.001, Mann–Whitney *U*-test. (**D**) Histological scores for dysplasia/neoplasia in mock and chronic cerulein treated K-Ras mutant mice. Detailed scoring criteria are described in *Methods*. Each symbol denotes data from one mouse. **, *P* < 0.01 (Mann–Whitney *U*-test).
(TIF)

S3 Fig Independent and overlapping bouts of pancreatic inflammation. (**A**) For independent bouts of inflammation, three acute cerulein pancreatitis events were induced two weeks apart, and inflammation and proliferation were assessed at the second (analysis time A) and third (analysis time C) bout of inflammation. HR was quantified 10 to 15 days after the last pancreatitis event. (**B**) For overlapping bouts of inflammation, three acute cerulein pancreatitis events were induced on days 1, 4 and 9. Inflammation and proliferation were assessed at the second (analysis time B) and third (analysis time D) bout of inflammation. HR was quantified 10 to 15 days after the last pancreatitis event. (**C**) Pancreas section from a control mouse shows healthy tissue. (**D,E**) Treatment with cerulein (both independent and overlapping) results in edema and an inflammatory infiltrate chiefly of neutrophils, indicating acute inflammation. (**F**) Ki-67 immunohistochemistry shows low levels of baseline proliferation in control pancreata. (**G**) Cell proliferation remains low in the pancreas during acute inflammation. (**H**) During regeneration from acute inflammation, Ki-67 positive nuclei appear, indicating regenerative proliferation. (**I**) Immunohistochemical detection of γH2AX phosphorylation in pancreas sections show low levels of DSBs in healthy pancreata. (**J**) During independent bouts of inflammation, nuclei with γH2AX foci (arrowhead) become apparent. (**K**) During overlapping bouts of inflammation, γH2AX positive nuclei are visible. (**C**-**E**) Original magnification, ×10. Scale bar = 200 μm. (**F**-**H**) Original magnification, ×20. Scale bar = 100 μm. (**I**-**K**) Original magnification, ×40.
(TIF)

S4 Fig Inflammation, proliferation and DSBs in independent and overlapping bouts of inflammation. Inflammation, cell proliferation and γH2AX foci formation were quantified in pancreas sections from mice treated with independent bouts of inflammation (blue bars) and with overlapping bouts of inflammation (purple bars). (**A,B**) Cerulein induces inflammation in both independent (n = 7) and overlapping (n = 8) treatment regimens. Severity of inflammation in control and cerulein-treated mice was quantified by a trained pathologist. (**C, D**) Quantification of nuclei positive for the proliferation marker Ki-67 shows no increase in independent bouts of inflammation (n = 7), and a large increase in overlapping bouts of inflammation (n = 8). (**E,F**) Quantification of nuclei positive for the DSB marker γH2AX (nuclei with >5 foci) shows a moderate increase in independent bouts of inflammation (n = 3), and no significant increase in overlapping bouts of inflammation (n = 3). Data are mean ± SEM. See *Methods* for detailed pathological scoring criteria. Statistical testing could not be performed in groups containing only zero values. * *P* < 0.05; ** *P* < 0.01, *** *P* < 0.001 (Student’s *t*-test).
(TIF)

S5 Fig Low-dose MNU treatment does not induce HR. Animals received MNU (7.5 mg/kg) in a single intraperitoneal injection, and HR was evaluated 3 to 5 weeks later. There is no significant difference between the numbers of fluorescent foci in control (n = 15) and MNU-treated (n = 14) mice. Symbols represent data from individual mice, horizontal bars show medians. ns, not statistically significant (Mann–Whitney *U*-test).
(TIFF)

S6 Fig Repeated intraperitoneal PBS injections have no effect on HR in the pancreas. Mice received single (*Left*, n = 85) or multiple (*Right*, n = 22) intraperitoneal PBS injections and the numbers of fluorescent foci in their pancreata were determined after *in situ* imaging as described in *Methods*. Symbols represent data from individual mice, horizontal bars show medians. ns, not statistically significant (Mann–Whitney *U*-test).
(TIF)
