# Supplementary figures and images for: Inflammation-Induced Cell Proliferation Potentiates DNA Damage-Induced Mutations In Vivo
Source: PLoS Genet. 2015 Feb 3;11(2):e1004901. doi: 10.1371/journal.pgen.1004901 (PMC4372043; doi:10.1371/journal.pgen.1004901)

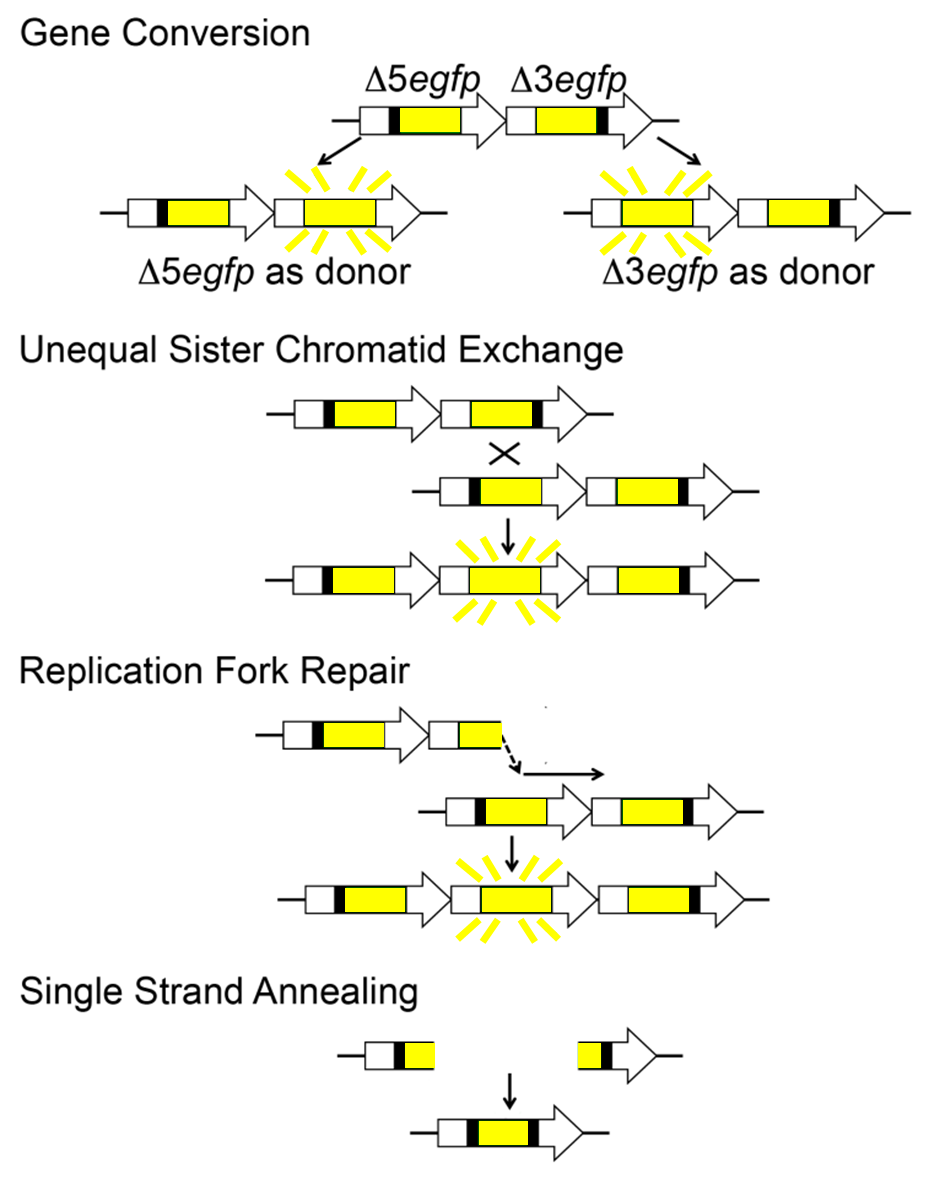

Supplement: S1 Fig — Each expression cassette is missing different essential EYFP coding sequences, such that neither is able to express functional protein. Gene conversion can lead to the transfer of sequence information from one cassette to the other, restoring full-length EYFP coding sequence and giving rise to fluorescence. Each cassette can be the donor or the recipient in a gene conversion event. The entire HR reporter is copied during S phase, making it possible for crossovers between sister chromatids (gene conversion with crossover) to reconstitute full-length EYFP. Note that a long tract gene conversion event would be indistinguishable. HR repair of a broken replication fork can also be detected using the FYDR substrate. The breakdown of a replication fork moving from left to right is shown. Reinsertion of the broken Δ3egfp end into the Δ5egfp cassette can restore full length EYFP. EYFP can analogously be restored by repair of forks moving in the opposite direction (not shown). Single strand annealing initiated by a DSB between the repeated cassettes can be readily repaired, but these events will not reconstitute full-length EGFP and thus SSA cannot be detected. (TIF) [file pgen.1004901.s001.tif]

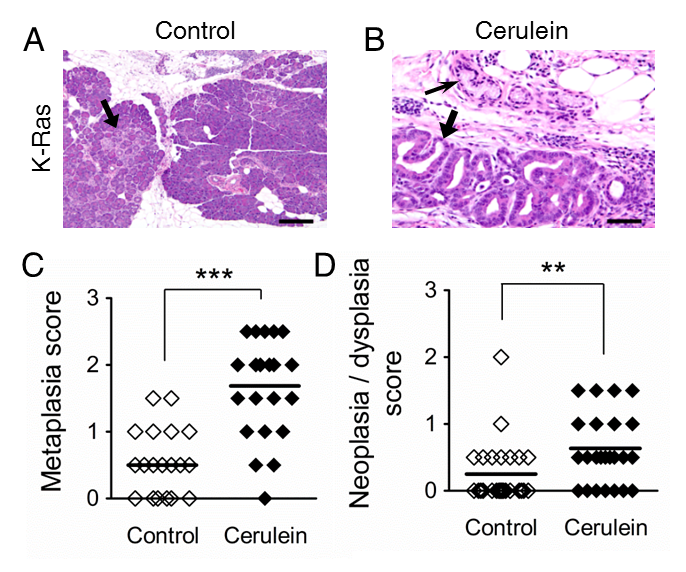

Supplement: S2 Fig — (A) Pancreas from mock treated K-Ras mutant mouse. Inflammation, acinar atrophy and interstitial fibrosis (arrow) are detectable. Acinar-to-ductal metaplasia is sparse. H&E staining. Original magnification, ×100. Scale bar = 160 μm. (B) Pancreas from K-Ras mutant mouse treated with chronic cerulein. Small focal proliferation of acinar tubules (thick arrow) with architectural and cytological atypia (dysplasia, low grade) surrounded by inflammation. Few acini with mucous metaplastic changes (thin arrow) are also present. Original magnification, ×400. Scale bar = 40 μm. (C) Histological scores for acinar-to-ductal metaplasia in mock and chronic cerulein treated K-Ras mutant mice. Detailed scoring criteria are described in Methods. Each symbol denotes data from one mouse. ***, P < 0.001, Mann–Whitney U-test. (D) Histological scores for dysplasia/neoplasia in mock and chronic cerulein treated K-Ras mutant mice. Detailed scoring criteria are described in Methods. Each symbol denotes data from one mouse. **, P < 0.01 (Mann–Whitney U-test). (TIF) [file pgen.1004901.s002.tif]

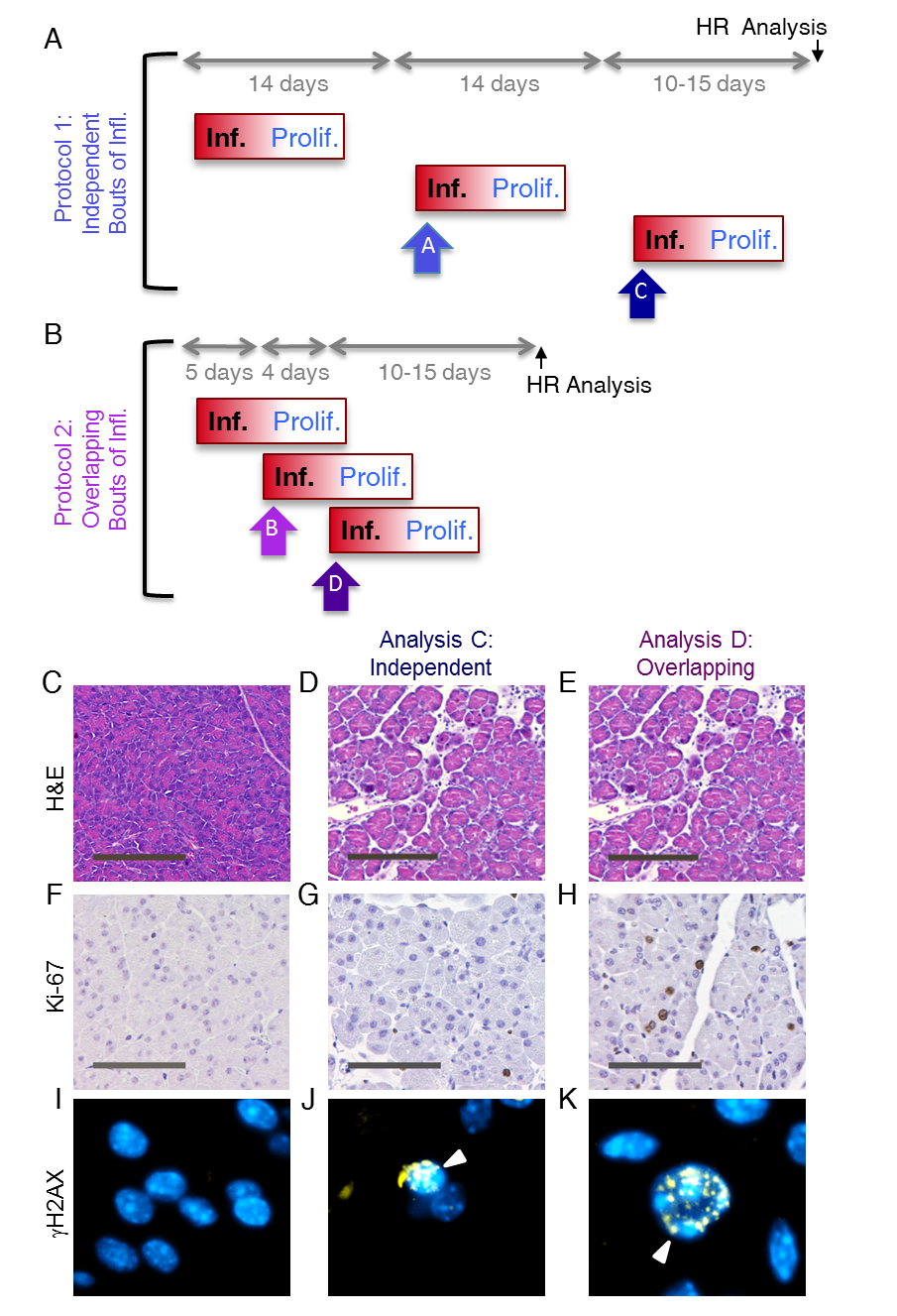

Supplement: S3 Fig — (A) For independent bouts of inflammation, three acute cerulein pancreatitis events were induced two weeks apart, and inflammation and proliferation were assessed at the second (analysis time A) and third (analysis time C) bout of inflammation. HR was quantified 10 to 15 days after the last pancreatitis event. (B) For overlapping bouts of inflammation, three acute cerulein pancreatitis events were induced on days 1, 4 and 9. Inflammation and proliferation were assessed at the second (analysis time B) and third (analysis time D) bout of inflammation. HR was quantified 10 to 15 days after the last pancreatitis event. (C) Pancreas section from a control mouse shows healthy tissue. (D,E) Treatment with cerulein (both independent and overlapping) results in edema and an inflammatory infiltrate chiefly of neutrophils, indicating acute inflammation. (F) Ki-67 immunohistochemistry shows low levels of baseline proliferation in control pancreata. (G) Cell proliferation remains low in the pancreas during acute inflammation. (H) During regeneration from acute inflammation, Ki-67 positive nuclei appear, indicating regenerative proliferation. (I) Immunohistochemical detection of γH2AX phosphorylation in pancreas sections show low levels of DSBs in healthy pancreata. (J) During independent bouts of inflammation, nuclei with γH2AX foci (arrowhead) become apparent. (K) During overlapping bouts of inflammation, γH2AX positive nuclei are visible. (C-E) Original magnification, ×10. Scale bar = 200 μm. (F-H) Original magnification, ×20. Scale bar = 100 μm. (I-K) Original magnification, ×40. (TIF) [file pgen.1004901.s003.tif]

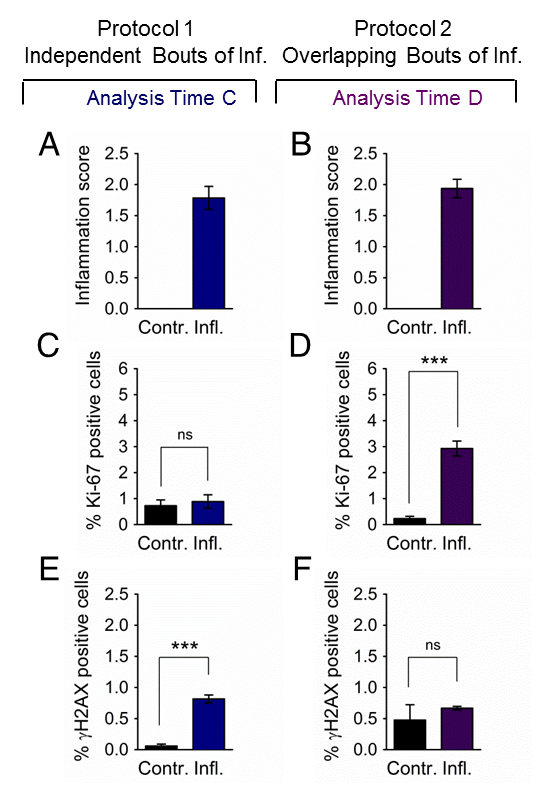

Supplement: S4 Fig — Inflammation, cell proliferation and γH2AX foci formation were quantified in pancreas sections from mice treated with independent bouts of inflammation (blue bars) and with overlapping bouts of inflammation (purple bars). (A,B) Cerulein induces inflammation in both independent (n = 7) and overlapping (n = 8) treatment regimens. Severity of inflammation in control and cerulein-treated mice was quantified by a trained pathologist. (C, D) Quantification of nuclei positive for the proliferation marker Ki-67 shows no increase in independent bouts of inflammation (n = 7), and a large increase in overlapping bouts of inflammation (n = 8). (E,F) Quantification of nuclei positive for the DSB marker γH2AX (nuclei with >5 foci) shows a moderate increase in independent bouts of inflammation (n = 3), and no significant increase in overlapping bouts of inflammation (n = 3). Data are mean ± SEM. See Methods for detailed pathological scoring criteria. Statistical testing could not be performed in groups containing only zero values. * P < 0.05; ** P < 0.01, *** P < 0.001 (Student’s t-test). (TIF) [file pgen.1004901.s004.tif]

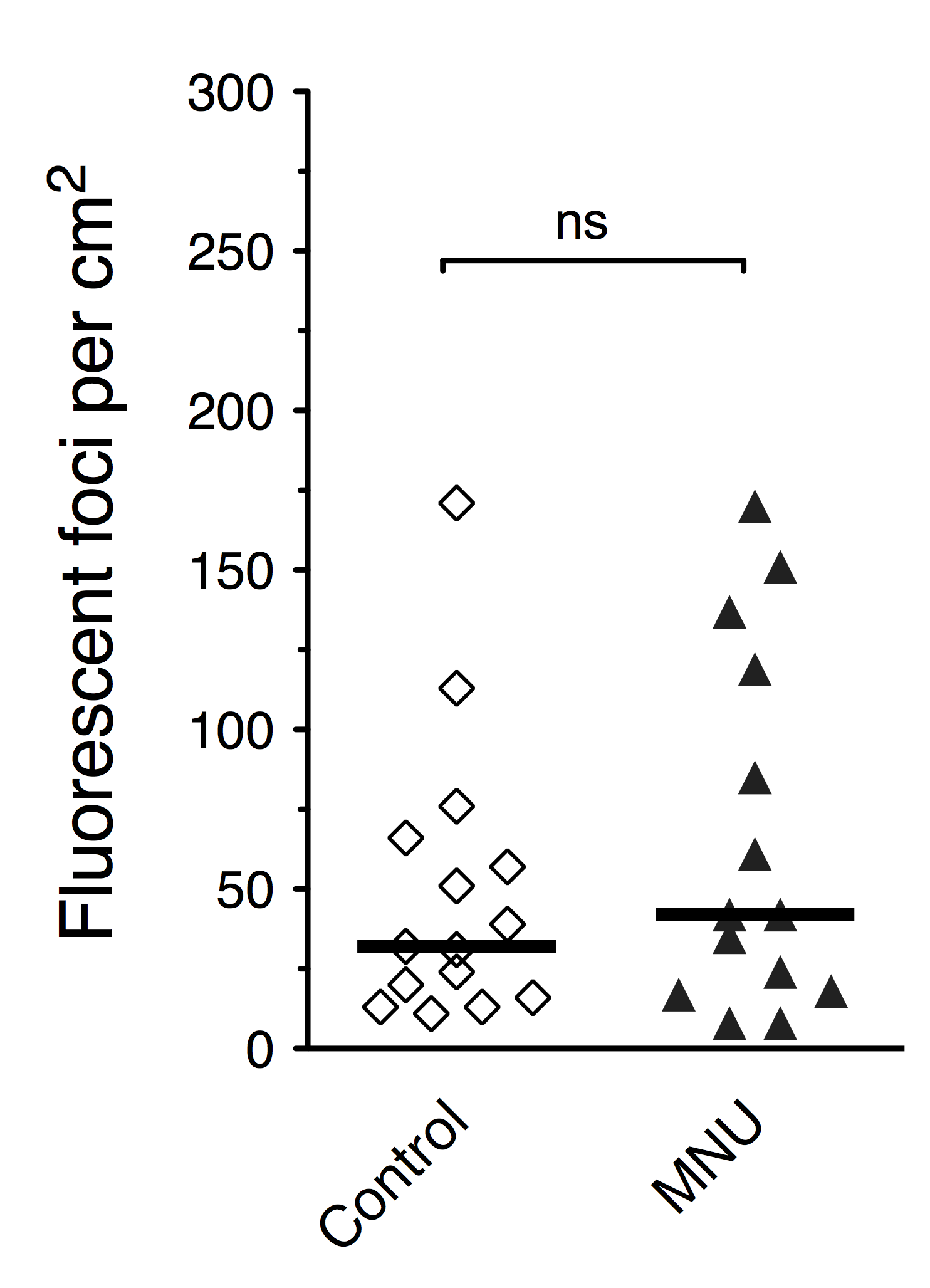

Supplement: S5 Fig — Animals received MNU (7.5 mg/kg) in a single intraperitoneal injection, and HR was evaluated 3 to 5 weeks later. There is no significant difference between the numbers of fluorescent foci in control (n = 15) and MNU-treated (n = 14) mice. Symbols represent data from individual mice, horizontal bars show medians. ns, not statistically significant (Mann–Whitney U-test). (TIFF) [file pgen.1004901.s005.tiff]

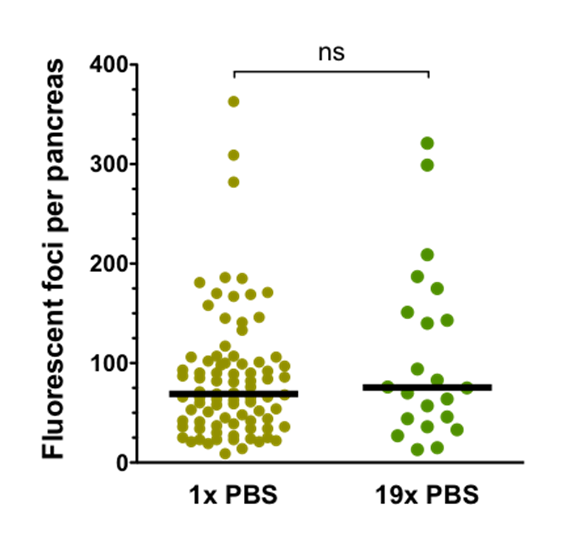

Supplement: S6 Fig — Mice received single (Left, n = 85) or multiple (Right, n = 22) intraperitoneal PBS injections and the numbers of fluorescent foci in their pancreata were determined after in situ imaging as described in Methods. Symbols represent data from individual mice, horizontal bars show medians. ns, not statistically significant (Mann–Whitney U-test). (TIF) [file pgen.1004901.s006.tif]
